# Mapping Immunity With Cutting‐Edge Spatial Biology and Tissue Cytometry Innovations

**DOI:** 10.1002/mco2.70775

**Published:** 2026-05-29

**Authors:** Lilibeth Cárdenas‐Piedra, Selwin G. Samuel, Felicitas Mungenast, Dinesh Yasothkumar, Rupert C. Ecker, Jyotsna Batra

**Affiliations:** ^1^ School of Biomedical Science Faculty of Health Sciences and Medicine Bond University Gold Coast Queensland Australia; ^2^ School of Biomedical Sciences Faculty of Health Queensland University of Technology Brisbane Queensland Australia; ^3^ Centre For Genomics and Personalised Health Queensland University of Technology Brisbane Queensland Australia; ^4^ ARC Training Centre For Cell and Tissue Engineering Technologies (CTET) Brisbane Australia; ^5^ School of Dentistry The University of Queensland Brisbane Queensland Australia; ^6^ Department of Oral Pathology and Microbiology Saveetha Dental College and Hospitals Chennai Tamil Nadu India; ^7^ TissueGnostics GmbH, EU Vienna Austria

**Keywords:** artificial intelligence, immunology, multiplexed imaging, multiplexed staining, spatial biology, tissue cytometry

## Abstract

The immune system operates within organized tissue landscapes, where spatial relationships between cells shape the nature and outcome of immune responses. Over the past decade, protein imaging‐based spatial technologies have transformed how immune responses are studied within their native tissue context, enabling simultaneous high‐plex detection of proteins and transcripts at single‐cell resolution. Driven by advances in computational methods such as tissue image cytometry, these approaches now allow quantitative extraction of spatial metrics such as cellular neighborhoods, cell–cell distances, coexpression patterns, and tissue architecture that are emerging as next‐generation spatial biomarkers of immune function and disease diagnosis and/or prognosis. This review provides a consolidated overview of immunology through the lens of imaging‐based spatial proteomics and tissue image cytometry, discussing the multiplex imaging platforms, staining strategies, and computational tools that enabled this shift. We survey applications spanning tumor immunology, autoimmune and infectious diseases, immunometabolism, neuroimmunology, and transplant immunology, revealing that immune organization is not random but spatially determined and directly linked to disease outcome. We further evaluate the strengths and limitations of current approaches and explore future directions including 3D imaging, artificial intelligence‐driven spatial analysis, and the clinical translation of spatial biomarkers.

## Graphical abstract

This review explores how spatial biology integrates multiplexed imaging and tissue cytometry to reveal immune organization within native tissue contexts.

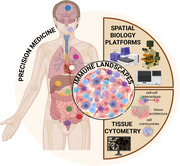

## 1 Introduction

The single‐cell technology revolution has transformed our understanding of biology by enabling the dissection of cellular identity at multiple molecular layers (genomics, transcriptomics, proteomics, and beyond). These technologies have uncovered molecular mechanisms that govern health and disease, allowing us to define what constitutes a cell and how cellular states change across conditions [1]. Yet a critical dimension remained missing: spatial context. Cells do not exist in isolation; they are embedded within tissues, where their function is shaped by interactions with the surrounding microenvironment. This recognition gave rise to spatial biology: the study of biomolecules and cells within their native tissue context [2, 3].

**FIGURE 1:**
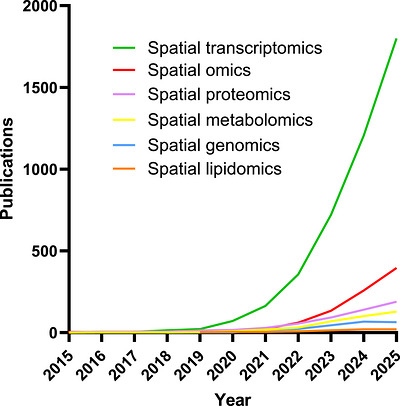
Spatial omics publications from 2015 to 2025. (PubMed database search: October 25, 2025; search queries: [“spatial transcriptomics,” “spatial omics,” OR “spatial multiomics” OR “spatial multiomics” OR “spatial multiomics,” “spatial proteomics,” “spatial metabolomics,” “spatial genomics,” “spatial lipidomics”]).

Over the past decade, spatial approaches have rapidly evolved into a rich ecosystem of modalities, initially led by spatial transcriptomics, and increasingly extending into full omics integration (Figure 1). These modalities are enabled by three broad classes of detection technologies: imaging‐based, sequencing‐based, and mass spectrometry (MS)‐based methods. Among spatial biology modalities, imaging‐based spatial transcriptomics and spatial proteomics, particularly antibody‐based spatial proteomics, have gained considerable traction, as these technologies build upon established diagnostic histopathology practices like in situ hybridization, immunohistochemistry (IHC) and light microscopy [4, 5]. By enabling simultaneous in situ detection of multiple targets—referred to as multiplex or high‐plex—spatial methods quantify not only cellular composition but also functional spatial biomarkers such as cell–cell distances, coexpression, cellular interactions, neighborhood organization, and tissue architecture [6]. As these technologies mature and become increasingly integrated into both research and clinical workflows, spatial biology now stands as one of the most dynamic frontiers in biomedical science.

Within this landscape, just as flow cytometry transformed cell suspension data into quantitative biological insight, spatial biology demands an equivalent analytical counterpart for tissue, and tissue image cytometry has emerged as that discipline, translating multiplex imaging data into interpretable, spatially resolved, and biologically meaningful information [7, 8, 9, 10, 11]. Of all domains where this framework finds application, spatial immunology has gained the most significant traction, driven largely by the growing interest in the tumor immune microenvironment (TIME) [12, 13, 14]. The TIME is highly heterogeneous, and while single‐cell approaches have uncovered novel mechanisms in which the TIME shapes cancer aggressiveness, metastasis, and therapeutic response, spatial analysis now provides the means to visualize and quantify these interactions directly within tissue architecture [6, 15, 16]. Importantly, the spatial frameworks shaped from studies in tumor immunology extend beyond oncology, offering a blueprint for studying the immune responses in a wide array of pathological contexts [13, 14].

As the field continues to mature, the challenge is shifting from generating spatial data to interpreting and translating it into biological and clinical knowledge. Central to this is the growing convergence between imaging‐based spatial biology and digital pathology, as tissue cytometry becomes increasingly routine in clinical settings. Despite the rapid progress, no comprehensive review has yet integrated the full analytical cycle with spatial immunology as a unifying thread [17, 18, 19].

This review navigates the core components of imaging‐based spatial biology and tissue image cytometry, with immunology as its central axis. We begin by examining the technological landscape and how it defines what can be measured and at what scale. We also address computational frameworks for tissue image cytometry and survey the biological applications of these methods across different disease contexts, highlighting how spatial frameworks are revealing shared organizational principles of immune responses across tissues and conditions. Finally, we discuss the current technical and analytical hurdles limiting the field, alongside emerging frontiers that will shape the next phase of this spatial odyssey.

## 2 Cutting‐Edge Technologies for Spatial Immune Profiling

Spatial immune profiling relies on a spectrum of technological innovations, from how targets are labeled in situ and visualized to how this information is extracted, quantified, and interpreted.

### 2.1 Probing the Spatial Landscape: Labeling, Detection, and Imaging

**FIGURE 2:**
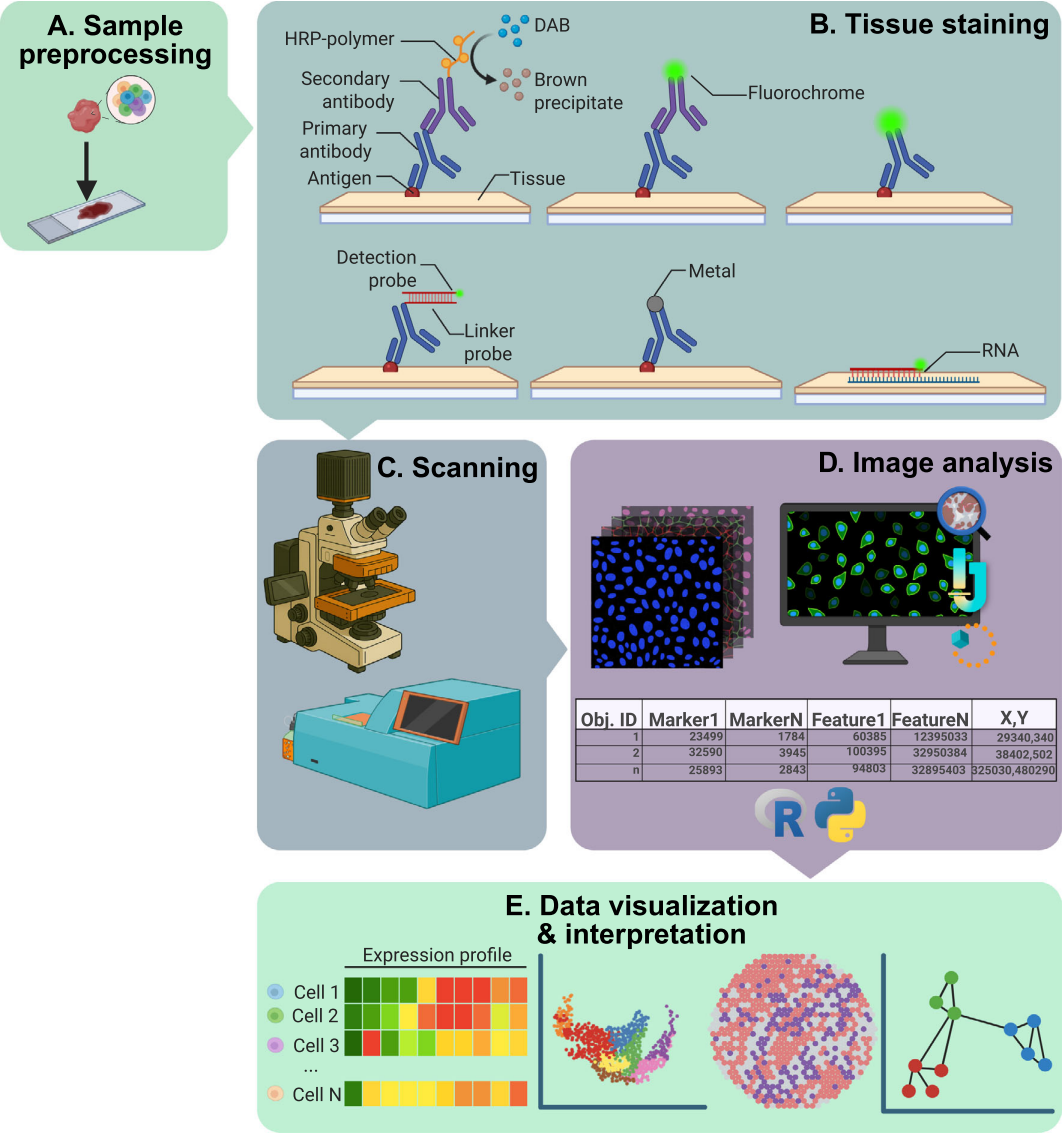
From tissue sections to spatial immune insights: (A) tissue samples are mounted on slides, (B) stained using antibody‐based methods for protein detection or RNA hybridization for transcript detection, and (C) scanned using advanced microscopy. In many commercial platforms, staining and scanning can be automated within a closed system. (D) The resulting images are preprocessed and analyzed with open‐source or commercial software. (E) Given the high dimensionality of the data, analysis typically begins with dimensionality reduction and clustering, followed by spatial analysis to assess cell neighborhoods, cell–cell interactions, and tissue organization, enabling improved visualization and interpretation of immune responses in situ (created with BioRender.com).

Tissue staining techniques are the basis of tissue image cytometry and spatial biology as they define how proteins or transcripts are labeled and subsequently imaged. Recent methodologies are built upon routinely used chromogenic IHC and RNA hybridization techniques (Figure 2). Table 1 summarizes the major staining techniques used in spatial biology, including their underlying principles.

**TABLE 1:** Tissue staining methods.

Target molecule	Staining method	Modalities	Principle	Multiplexing	Product name	References
Protein	Chromogenic IHC	Conventional (indirect)	Enzyme‐linked secondary Ab produces a colored precipitate in presence of a substrate.	Not suitable, limited to six chromogens due to color overlap	DISCOVERY Multiplex (Roche)	[20]
	Fluorescence IHC (immunofluorescence, IF)	Conventional (direct/indirect IF)	Direct/indirect Ab‐fluorochrome conjugation	CycIF (requires signal removal between cycles)	ChipCytometry (Canopy Biosciences), seqIF (Lunaphore)	[21]
		Tyramide signal amplification (TSA)	HRP‐linked Ab catalyzes tyramide‐conjugated fluorochrome deposition.	Permanent signal, limited to spectral overlap	Opal Multiplex Detection kits (Akoya Biosciences), TSA Multiplex IHC Assay Kits (TissueGnostics)	[22]
		Oligo‐barcoding (direct/indirect)	Oligo‐conjugated Ab hybridizes with an oligo‐conjugated fluorochrome.	Sequential fluorochrome‐conjugated detection probe hybridization (requires signal removal between cycles)	PhenoCycler (Akoya Biosciences) InSituPlex (Ultivue)	[23]
	IMC	Mass‐tagged antibody	Metal‐linked Ab conjugation; metal is detected by laser ablation and TOF‐MS.	All‐at‐once staining	Maxpar (Standard Biotools)	[24]
RNA	Fluorescence in situ hybridization (FISH)	Conventional	Fluorochrome‐conjugated probe hybridized directly with target RNA.	Weak sensitivity and nonreversible binding	N/A	[25]
		RNAscope	Intermediate oligonucleotides hybridize RNA targets to enhance detection sensitivity. Fluorochrome‐conjugated probes then bind to the intermediates for signal visualization.	Sequential fluorochrome‐conjugated detection probe hybridization (requires signal removal between cycles)	RNAscope HiPlex (Biotechne)	[26]
		Rolling circle amplification			RNAsky	[27]
		Combinatorial barcoded sequential hybridization			MERFISH	[28]
		Branched probe hybridization			N/A	[29]

*Abbreviations*: Ab: antibody; HRP: horseradish peroxidase; MERFISH: multiplexed error‐robust fluorescence in situ hybridization; seqIF: sequential Immunofluorescence.

Improvements in staining methods, such as tyramide signal amplification (TSA) [30, 31], cyclic immunofluorescence [32], or multiplexed error‐robust fluorescence in situ hybridization [28], have significantly increased multiplexing capabilities. These advances now enable the detection of dozens of proteins or transcripts within the same tissue section.

Pioneering work by Steiner et al. [9] introduced a methodology integrating multiplex immunofluorescence staining, automated scanning, and advanced image cytometry to quantify tissue‐resident leukocytes in human renal carcinoma tissues. This early approach demonstrated that complex immunophenotyping can be conducted directly on tissue sections.

**FIGURE 3:**
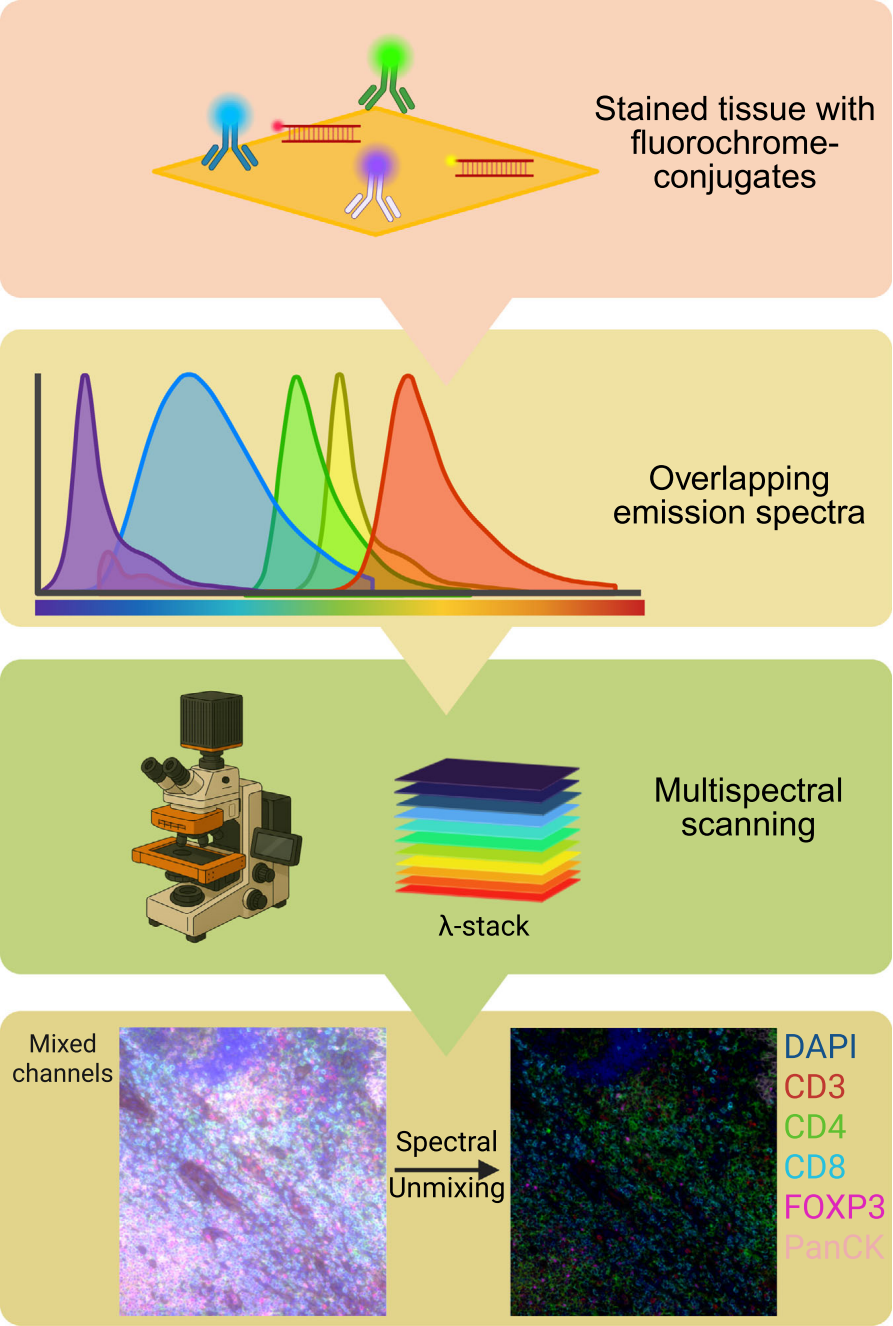
Principle of multispectral imaging for high‐plex tissue analysis. Tissue sections are stained with fluorochrome conjugates to detect the target structures of interest. When multiple fluorophores are used simultaneously, their emission spectra often overlap, making it difficult to specifically detect each signal. Detector crosstalk, however, will create false‐positive events in spectrally adjacent channels. LCTF in multispectral scanners allow the acquisition of images across narrow wavelength bands, generating what is known as a λ‐stack (lambda stack). This process captures the full emission profile of all fluorochromes across the spectrum in all pixels. Computational spectral unmixing compares the emission profile from a sample to reference spectra and then separates these overlapping spectra by a technology referred to as “linear unmixing” into distinct channels, generating clean, marker‐specific images (created with BioRender.com).

Since then, tissue staining and image cytometry techniques have been widely applied to explore the pathobiology of numerous diseases [33, 34, 35, 36]. These methods have evolved into a broad ecosystem of commercial platforms designed for multiplexed spatial profiling, ranging from multispectral imaging systems to fully integrated platforms that combine automated multiplexed staining with imaging in a single workflow (see Table 2). In the latter, tissue staining is commonly performed using microfluidic‐based cyclic labeling systems. Imaging relies on fluorescence microscopy, using fluorochrome‐conjugated antibodies or oligonucleotides. However, the conventional approach is constrained by spectral overlap, which limits how many markers can be visualized per staining round. To overcome these limitations, platforms such as the PhenoImager HT (Akoya Biosciences) employ multispectral imaging. This microscopy modality allows more fluorochromes to be used simultaneously, increasing the multiplexing capacity. In practice, this is achieved through technical advances in multispectral scanners such as the incorporation of liquid crystal tunable filter (LCTF) technology like in TissueFAXS spectra (TissueGnostics) multispectral scanner, along with spectral unmixing algorithms (see Figure 3) [37, 38, 39].

**TABLE 2:** Image‐based spatial technologies.

Technology/vendor	Target/Staining method	Probe ecosystem	Signal removal	Imaging	Sample	Automation	Analysis	Bio‐formats compatibility	Advantages	Limitations	References
CellDIVE/Leica Microsystems	Protein: Direct IF‐CycIF	Leica's validated primary Abs library (Cell Signaling); can validate your own	Chemical bleaching	Widefield fluorescence Up to 20×	FFPE	Walk‐away image acquisition	Image viewing software for stitching and registration Aivia	Partial	Staining can be automated by coupling to BioAssembly Bot 200. Up to 4 proteins detected per staining round Permits small number of primary‐secondary Ab pairs for indirect IF	No application for FF samples	[40]
CellScape/Canopy Biosciences	RNA: FISH/RNAscope Protein: Direct IF—CycIF (ChipCytometry)	Off‐the‐shelf Abs, database validated antibodies, or VistaPlex Assay Kits optimized and validated	Filtered photobleaching (EpicIF technology)	Epifluorescence microscope; 182 nm/pixel	FF, FFPE, cells	Fully automated closed platform; requires manual exchange of reservoirs to expand total probe number when run out the reservoir of total 75 probes	CellScape App. HORIZON Viewer software	Partial	Five probes/cycle/reservoir, 15 reservoirs (total 75 Abs per run); reservoirs can be exchanged to increase plex. Samples loaded in safe chip to preserve them	Up to 4 samples per run	[33]
COMET/Lunaphore	RNA: FISH/RNAscope (RNAscope HiPlex Pro) Protein: indirect IF‐CycIF (seqIF)	SPYRE amplification kit by Lunaphore, Lunaphore recommended antibodies, custom antibodies RNAscope probe catalog by ACD (Biotechne) or custom design	Cleavage of fluorescence signal in RNA detection Elution of the primary and secondary antibodies and quenching in protein detection	Fluorescence. 0.28 µm/pixel	FF, FFPE	Fully automated staining, imaging, and signal removal cycles, closed platform; up to four slides per run	Software for image viewing and evaluation like background extraction; integrated with HORIZON by Lunaphore	Full	Samples can be re‐used for downstream applications. Z‐stack imaging to increase RNA signal detection Optimized secondaries: Alexa Fluor Plus 488, 555, 647, or 750	Manual sample preprocessing Proteomics: 40‐plex per automated run Multiomics: 12 RNA plus up to 12 protein or 4 RNA plus up to 28 proteins	[41]
CosMx/Bruker	RNA: FISH /branched probe hybridization Protein: oligobarcoding‐sequential probe detection hybridization	Library of validated RNA probes and oligonucleotide conjugated antibodies; can validate your own	UV cleavage of fluorochromes	Widefield microscope; 0.1–0.5 µm/pixel	FF, FFPE	Automated reporter hybridization, imaging, and signal removal	AtoMx Spatial Informatics Platform for data analysis or custom pipelines	Partial	Up to four slides per run Cloud‐based storage and analysis platform Up to 6000 RNA and 64 validated protein analytes	Manual preprocessing and staining Protein detection is limited to morphology markers.	[42, 43]
EVOS S1000/ThermoFisher Scientific	RNA/protein: any transmission and/or fluorescence‐based method	ThermoFisher Scientific's library of conjugated primary antibodies; can validate your own Aluora Spatial Amplification kits	N/A	Brightfield and multispectral; up to 40×	FF, FFPE	Automated image acquisition	N/A	Full	Channels auto‐configuration after dye selection Up to 9‐plex in a single round	Manual sample preprocessing and staining	[44]
Hyperion XTi imaging System/ Standard Biotools	RNA: FISH/ RNAscope (RNAscope HiPlex V2) Protein: mass‐tagged antibody (Maxpar)	IMC panels, Maxpar antibodies, Maxpar IMC cell segmentation kit, and custom antibodies requiring conjugation (Maxpar labeling kits or custom conjugation services)	Epifluorescence (only for overview image)	Image reconstruction according to abundance of each isotope at each ablation spot	FF, FFPE	Automated detection and imaging	Software for image acquisition, viewing, and tissue mode analysis (CyTOF software and MCD SmartView)	Partial	Slide loader (up to 40 slides); no autofluorescence or spectral overlap as no light emission is measured.	Manual preprocessing and staining; cost	[24, 45]
MACSima/Miltenyi Biotec	RNA: FISH/rolling circle amplification (RNAsky) Protein: Direct IF‐CycIF (MICS)	Miltenyi Biotec's REAfinity, REAdye/lease antibodies, and RNAsky probes	Photobleaching for REAfinity abs or fluorochrome removal from REAdye/lease Abs or RNAsky probes	Widefield epifluorescence; up to 20×	FF, FFPE, cells	Walk‐away staining and image acquisition	MACS iQ View	Partial	Proprietary antibody chemistries	Manual sample preprocessing	[46, 47]
MERSCOPE/Vizgen	RNA: FISH/combinatorial barcoded sequential hybridization (MERFISH) Protein: indirect oligobarcoding and sequential probe detection hybridization	MERFISH probes; MERSCOPE Protein Stain Reagent Kits (up to 6 oligo‐conjugated secondary antibodies)	Photobleaching	Fluorescence. 100 nm/pixel	FF, FFPE, cells	Walk‐away image acquisition	Integrated MERSCOPE Visualizer and open‐source Vizgen postprocessing tool	Partial	High resolution	No brightfield imaging Manual sample preprocessing Manual objective switch from preview to acquisition Limited protein detection Limited to 10 regions and max total acquisition area of 1 cm^2^; 1 MERSCOPE slide capacity	[43, 48, 49]
Orion/RareCyte	Protein: Direct IF; no cycling	Rarecyte's validated primary Abs library. Optimized ArgoFluor dyes for one‐shot immunostaining Can validate your own	N/A	Brightfield and multispectral; up to 20×	FF, FFPE	Walk‐away image acquisition	On‐board software for image viewing and quantitation	Full	Can image 20 channels per round	Manual sample preprocessing and staining Up to two slide capacity	[50]
PhenoImager Fusion/ Akoya Biosciences	Protein: oligobarcoding/sequential probe detection hybridization	PhenoCode antibody panels; Akoya's primary validated Abs library; can validate your own	Removal of fluorochrome‐conjugated probes	Brightfield and multispectral up to 0.25 µm/pixel	FF, FFPE	Automated staining when using PhenoCycler instrument; walk‐away image acquisition; two slides per run	Phenochart; inForm; phenoptrReports	Full	Separates up to seven colors	Up to four slide capacity	[51]
PhenoImager HT/Akoya Biosciences	RNA/protein: any transmission and/or fluorescence‐based method	Akoya's validated primary antibodies library; can validate your own Opal Multiplex IHC Kits (TSA using Opal fluorochromes)	N/A	Brightfield and multispectral Up to 40×, 0.25 µm/pixel	FF, FFPE	Walk‐away image acquisition	Phenochart; inForm; phenoptrReports	Full	Parallel scanning and unmixing 80 slide capacity with continuous slide loader Multispectral separates up to nine colors	Manual sample preprocessing No staining capability	[52]
TissueFAXS Spectra SL/TissueGnostics	RNA/protein: any transmission and/or fluorescence‐based method	N/A	N/A	Brightfield and multispectral; up to 100×	FF, FFPE	Walk‐away image acquisition	TissueFAXS viewer; StrataQuest	Full	Slide loader capacity of 120 slides	Manual sample preprocessing and staining	[53]
Xenium/10xgenomics	RNA: FISH/rolling circle amplification Protein: oligobarcoding and sequential probe detection hybridization	Ready‐to‐use RNA panels; custom RNA panels Xenium protein subpanels	Removal of fluorochrome‐conjugated probes	Fluorescence. 0.2 µm/pixel	FF, FFPE	Automated sequential fluorescent probe hybridization, imaging, and signal removal	Xenium Onboard Analysis; Xenium Ranger; Xenium Explorer	Partial	Up to 5000‐gene panels Nondestructive	Multiomics function is limited to be used with <500 gene panels. Limited protein detection to Xenium protein subpanels (max 27 proteins) No automated sample preprocessing, probe hybridization or antibody staining	[43]

*Abbreviations*: FFPE: formalin‐fixed paraffin embedded; MICS: MACSima imaging cyclic staining; FF: fresh frozen; MERFISH: multiplexed‐error robust fluorescence in situ hybridization; CyTOF: cytometry by time of flight; UV: ultraviolet; N/A: not applicable.

Going beyond fluorescence, imaging mass cytometry (IMC) employs metal‐conjugated antibodies detected via time‐of‐flight MS, eliminating spectral overlap. The image is reconstructed pixel by pixel based on metal abundance, maintaining both resolution and multiplexing power [54].

### 2.2 From Images to Insights: Tissue Image Cytometry for Spatial Analysis

Most spatial biology platforms provide built‐in image processing capabilities, but these are largely limited to basic visualization and technology‐specific preprocessing tasks, including image registration, stitching, and alignment. While such functions are necessary for the initial handling of spatial imaging data, they do not typically support the breadth of analyses required for deeper characterization. Researchers therefore frequently rely on third‐party software tools or bespoke pipelines, often implemented in R or Python, to enable more advanced analytical workflows (see Table 3).

**TABLE 3:** Open‐source and commercial tissue image cytometry tools.

Category	Name	Open source	Developer/Vendor	Core functionalities	Bio‐formats integration	Implementation	References
Advanced segmentation tools (ML/DL)	CellPose	Yes	Stringer et al. [55]	Whole cell segmentation; allows custom fine‐tuning and integrates restoration for noisy/blurry images	No	Python; GUI	[56]
	CellSeg	Yes	Lee et al. [57]	Nucleus segmentation, expanded cell mask, and single cell statistics (coordinates and marker intensities)	No	Python (Jupyter Notebooks)	[58]
	Mesmer	Yes	Greenwald et al. [59]	Whole cell segmentation	No	Web app	[60]
	StarDist	Yes	Schmidt et al. [61]	Whole cell segmentation	No	Python; available as plugins for other software (ImageJ/Fiji, Napari, QuPath, Icy, KNIME)	[62, 63, 64]
	UnMICST	Yes	Yapp et al. [65]	Three models for nuclei segmentation	No	Python; integrated into MCMICRO workflow	[66]
Image visualization tools	Napari	Yes	Sofroniew et al. [67]	Interactive viewer, supports annotation, and plugin‐based image analysis workflows	Yes	Python; GUI	[68]
	TissUUmaps	Yes	Pielawski et al. [69]	Interactive viewer, supports overlay of expression data, segmentation masks, and annotations for exploratory analysis	No	Web app; desktop app	[70]
Integrated analysis suites (GUI)	Cell Profiler/Cell Profiler Analyst	Yes	Stirling et al. [71]	Cell segmentation, quality control ML for cell phenotyping, optimized for small areas rather than full tissue sections or TMA slides	Yes	Python‐based GUI	[70, 72, 73, 74]
	HALO	No	Indica Labs	Interactive visualization, image preprocessing, classifiers for tissue masks, segmentation, phenotypes, and artifacts (HALO AI); integrated features as a Highplex FL module supporting multiplexed image analysis; apps available	No	Desktop app	[64, 75, 76, 77, 78]
	histoCAT	Yes	Schapiro et al. [79]	Image visualization, dimensionality reduction, cell phenotyping using PhenoGraph, spatial analysis (neighborhood analysis)	No	Available as MATLAB app or stand‐alone executable requiring free MATLAB Runtime	[60, 80, 81]
	Ilastik	Yes	Berg et al. [82]	Interactive ML platform for segmentation, tracking user‐provided annotations, pixel classification, object classification	No	Desktop app; available as a Fiji plugin	[83]
	inForm	No	Akoya BioSciences	Image visualization, spectral unmixing, cell segmentation, tissue classifier, cell phenotyping, exports cell‐level data for downstream analysis (Phenoptr, QuPath)	No	Desktop app	[39]
	Phenoplex	No	Visiopharm	Interactive visualization, cell segmentation, tissue classifier, unsupervised clustering for cell phenotyping or manual gating, spatial analysis (cell neighborhood, distances), phenotype heatmaps; apps available	No	Desktop app	[84]
	QuPath	Yes	Bankhead et al. [85]	Interactive visualization, cell segmentation, pixel‐based classifier, distance measurement	Yes	Desktop app	[63, 64, 75]
	StrataQuest	No	TissueGnostics	Interactive visualization, image preprocessing, spectral unmixing, AI‐powered nuclei segmentation, identified cell masks, tissue classifier, dimensionality reduction, spatial analysis (distance, proximity); AI center/deep learning training environment; 80+ Apps available	Yes	Desktop app	[86, 87]
Spatial omics frameworks	CytoMAP	Yes	Stoltzfus et al. [11]	Spatial analysis toolkit implementing spatial statistics for quantifying tissue architecture, cell neighborhoods, and interactions, with tools for gating, clustering, and multidimensional visualization	No	MATLAB‐based GUI	[88, 89]
	Scanpy/squidpy	Yes	Palla et al. [90]	Dimensionality reduction, unsupervised clustering for cell phenotyping, clustering	No	Python	[62, 91]
	SpatialData	Yes	Marconato et al. [92]	Framework for standardized storage, access, and integration of spatial omics data (images, masks) across technologies	No	Python	[68, 93]
Workflows	Cecelia	Yes	Schienstock et al. [94]	Interactive visualization, cell segmentation, and annotation (gating, clustering), spatial analysis (neighborhoods, regions, interactions)	Yes	Hybrid R/Shiny and Python/napari; can be run on high‐performance computing systems; available as R package or via Docker	[95]
	MCMICRO	Yes	Schapiro et al. [96]	Containerized workflow for image preprocessing (illumination correction, alignment, stitching), TMA core detection (Coreograph), segmentation (UnMICST or Se3gmenter), single‐cell features (MCQuant), quality control (CyLinter), spatial analysis (SCIMAP) and visualization (Minerva)	Yes	Implemented in Nextflow and Galaxy; uses Docker/Singularity containers	[25]
	SPACEc	Yes	Tan et al. [97]	Tissue detection, cell segmentation, data preprocessing and normalization, cell‐type annotation, data exploration, and spatial analysis (cellular neighborhoods, interface, interaction, and proximity analysis)	No	Python	[98]

*Abbreviation*: GUI: graphical user interface

Open‐source software and tools benefit from large and active user communities, online documentation, and forums that facilitate troubleshooting and the sharing of novel workflows. They favor flexibility and the creation of highly customized pipelines tailored to specific biological questions as most researchers combine multiple software within a single project [49, 99, 100, 101]. However, the same reliance on community‐driven development means that quality control is largely based on “in the field” testing by users, with issues and bugs identified during scientific work rather than through formal validation. Further, while their modularity enhances innovation and adaptation, at the same time it introduces variability and technical barriers, limiting reproducibility and comparability, which is particularly important when looking toward clinical adoption [102].

Popular open‐source software tools include ImageJ [103], QuPath [85], or CellProfiler [104]. Open‐source tools are widely adopted within the academic community due to their accessibility and flexibility for creating customized analysis pipelines [49]. On the other hand, commercial software solutions offer robustness and regulatory compliance, but at the expense of flexibility and cost. While image file formats across platforms are not yet fully standardized, limiting data exchange between tools, there is a growing trend toward open and interoperable formats. For imaging‐based spatial proteomics, OME‐TIFF has emerged as a community standard, while for imaging‐based spatial transcriptomics, frameworks such as SpatialData aim to unify image and molecular data into a single interoperable structure [92, 105]. Table 3 summarizes popular software and tools for tissue cytometry in the spatial analysis field. A key strength of tissue cytometry software lies in its ability to extract a broad range of cellular features, from marker expression and morphology to spatial relationships between cells.

The development of spatial metrics has been particularly transformative, enabling quantitative assessment of tissue architecture and immune organization, which can be used as predictive spatial biomarkers [6]. A notable example is the TIME Spatial score, which uses the spatial distribution of five biomarkers to predict cancer recurrence risk within 5 years of surgery in hepatocellular carcinoma patients [106].

As multiplexing grows and images contain hundreds (as in spatial proteomics) to thousands (as in spatial transcriptomics) of markers per sample, each individual cell becomes a data point with high‐dimensional biological features [107]. Such depth is invaluable for research, allowing discovery of novel immune subsets, functional states, and spatial interactions that otherwise remain hidden. However, it also necessitates specialized analysis frameworks to decipher the statistical complexity of such high‐dimensional data, mostly implemented in Python or R like the scverse ecosystem—an integrated toolset for single‐cell and spatial omics analysis [51, 90, 92, 108]. These frameworks support more powerful statistical methods like spatial statistics or dimensionality reduction (e.g., PCA, t‐SNE, UMAP, or SONG), which compress high‐dimensional features into 2D or 3D spaces where we can easily visualize patterns and clusters that would otherwise be hidden [109, 110].

Equally important is the ability to directly visualize analytical results on tissue images, allowing researchers to cross‐validate computational outputs with the actual histological context. This advantage is particularly evident in platforms that offer intuitive graphical user interfaces, which facilitate both exploration and quality control of spatial data.

Advances in artificial intelligence (AI), particularly in deep learning (DL), are increasingly integrated into tissue cytometry and histopathology [111]. These tools automate tasks such as immune cell identification [112], biomarker scoring (e.g., PD‐L1) [113], and even tertiary lymphoid structures (TLS) identification [114], in routine histological stains like hematoxylin and eosin (H&E) or chromogenic IHC.

More recently, AI‐based approaches have been employed to extract features from raw images or high‐dimensional datasets generated by spatial platforms, taking advantage of the vast number of features available, to better predict cell phenotypes [115], perform region classification [116], and even support clinical diagnostics [117].

Some platforms have begun integrating AI‐driven modules directly. For instance, open‐source software like QuPath, or commercial like StrataQuest offer iterative training and prediction models. However, AI‐based advanced tools are mainly available in the form of code repositories, like CellLens, a DL‐based pipeline that uniquely combines single‐cell expression profiles, spatial coordinates, and image features to classify immune populations [118]. However, the growing complexity of AI‐driven and multimodal analyses also highlights the need for robust standards in data handling and image analysis. Without interoperable and reproducible frameworks, the integration of imaging data with other molecular modalities remains technically challenging.

Community‐driven efforts for standardization in image analyses like the *Quality Assessment and Reproducibility for Instruments & Images in Light Microscopy* initiative [119], the FAIR Data Principles spearheaded by the Global BioImaging consortium [120], or the Spatio‐Temporal OMICS Consortium play a crucial role in developing a data ecosystem that allows imaging data to be interlinked with other modalities and facilitate a comprehensive understanding of biological processes as cellular basis of diverse medical conditions in immunology and beyond.

## 3 Immunity in Context: Spatial Applications to Understand Pathogenesis

Across diverse pathological contexts, including cancer, tuberculosis (TB), and rheumatoid arthritis (RA), a unifying principle has become increasingly evident: immune responses are spatially structured rather than diffusely distributed. Features such as granulomas, perivascular niches, immune exclusion zones, and TLSs are not incidental histological correlates, but organized tissue architectures that both reflect and regulate local immune activity. Accordingly, cellular function cannot be inferred solely from phenotype, as it is also conditioned by spatial localization within the tissue context. Macrophages residing in the granuloma core, for instance, may express markers similar to those at the periphery, yet be subject to distinct cellular interactions, signaling gradients, and metabolic constraints that confer nonequivalent functional roles. Spatially resolved approaches make these relationships measurable, enabling the identification of conserved spatial patterns that contribute to disease pathogenesis across tissues and clinical contexts.

### 3.1 Tumor Immunology

Studying the TIME has become one of the most prominent applications of spatial biology over the past decade [16]. In the era of immunotherapies, where treatment success depends on the dynamic interplay between tumor and immune cells, spatial biology provides a powerful framework to visualize, quantify, and interpret these relationships, revealing spatial patterns predictive of treatment response that may outperform current biomarkers [121, 122, 123].

It is increasingly evident that tumor infiltrating lymphocytes and other cell types are organized into structured spatial neighborhoods. Dissecting the composition and status of these cellular communities could help in developing more targeted immunotherapy strategies. Liu et al. [124] identified fibroblast‐centered niches conserved across 10 cancer types. These niches arose from the interactions between cancer‐associated fibroblasts and neighboring tumor, stromal, and immune cells, with distinct spatial arrangements correlating with different patient prognoses. Garcia‐Vicien et al. [125] used multiplexed imaging to uncover a highly immunosuppressive microenvironment in nonencapsulated colorectal cancer liver metastases compared with encapsulated ones, underscoring not only the diagnostic relevance of histological architectures but also the importance of assessing the immune context to better understand therapy responses.

Among the key drivers of antitumor immunity are CD8+ T cells. Many immunotherapies aim to increase cytotoxic CD8+ T cell infiltration and spatial biology helps reveal whether these cells are not only present but also properly positioned [126, 127, 128]. For example, spatial analysis of CD8+ T cell distribution in breast cancer has shown that distance‐based metrics such as proximity and consistency, which capture how close and how uniformly CD8+ T cells are positioned relative to tumor cells, are more prognostically relevant than quantity alone [12, 129]. A similar principle is applied to other cancers. For example, the proximity of CD8+ tissue‐resident memory T cell subsets to tumor cells is dynamically altered during tumor progression in lung cancer, with reduced interactions between specific CD8+ subsets and tumor cells reflecting immune evasion [130].

Spatial approaches are also used to monitor immune remodeling following therapy [131]. For instance, it has been observed that therapeutic vaccination in prostate cancer resulted in distinct T cell infiltration patterns in different tumor compartments [132].

Collectively, tumor immunology has laid the conceptual foundation for spatial biology, demonstrating how immune cells organize into functional architectures and how these patterns correlate with outcomes [15]. T cell exclusion from tumor cores, fibroblast‐centered niches shaping immune access, and the prognostic weight of the tumor‐stroma boundary are not unique to cancer. Analogous structures appear across inflammatory, infectious, and degenerative contexts, suggesting that the spatial logic of immune organization may be a fundamental principle of tissue biology rather than a cancer‐specific phenomenon.

### 3.2 Autoimmune and Allergic Diseases

Autoimmune and allergic conditions arise when the immune system reacts to self‐antigens or harmless environmental stimuli. These disorders often involve complex immune–stromal interactions, but their precise etiology remains unclear in many cases. Here we highlight some examples where spatial proteomics has provided novel insights into the microenvironment organization underlying these conditions.

#### 3.2.1 Rheumatoid Arthritis

RA is a chronic autoimmune disease characterized by the production of autoantibodies to citrullinated proteins and inflammation in the synovial tissue. Single‐cell studies have greatly enhanced our understanding of synovial cellular heterogeneity, but most of this research relied on dissociated tissue, losing spatial context [133, 134, 135, 136, 137].

A spatial study using IMC by De Lima et al. [24] identified a macrophage subset (LYVE1+) localized around blood vessels that reappears in remission after treatment, suggesting an immunomodulatory role maintaining tissue homeostasis. In parallel, another study by Richard et al. [138] identified perivascular collagen VI as a key structural and functional niche that regulates immune responses in arthritis. Together, these findings highlight the perivascular niche as a regulatory hub in RA pathogenesis. Notably, the perivascular niche is not unique to autoimmunity, as in cancer it has similarly been identified as a hub where immune evasion and metastatic behavior are regulated [139, 140].

#### 3.2.2 Allergic Disease

Allergic diseases also feature chronic inflammation, often driven by dysregulated immune–epithelial and immune–stromal interactions. Conditions like asthma or atopic dermatitis are shaped by inflammatory niches within the skin, airways, and mucosa [141, 142].

Spatial approaches highlight how allergic inflammation is sustained by organized immune niches. For instance, in a mouse model of house dust mite‐induced asthma, He et al. [143] combined multiple spatial technologies, including multiplexed RNAScope, multiplex immunofluorescence, and spatial transcriptomics, and observed that IL‐2‐rich niches in mediastinal lymph nodes (those draining the thoracic cavity and adjacent to the lungs) drive differentiation of migratory CD4+ T cells which then home to the lungs and sustain allergic response. Crucially, emerging evidence suggests that this spatially organized priming does not end in the lymph node, as the formation of pulmonary TLSs in the allergic lung could serve as progenitor niches where allergen‐specific T cell populations are maintained and replenished, sustaining chronic inflammation [144].

### 3.3 Infectious Diseases

Mapping host–pathogen interactions in situ provides a deeper understanding of how immune responses evolve within infected tissues and how these responses influence disease progression.

#### 3.3.1 Tuberculosis

TB remains the leading cause of death from a single infectious agent. In TB, immune cells aggregate into granulomas in the lung, but the functional heterogeneity and evolution of these structures are still poorly understood [145].

Multiplex imaging has revealed substantial immune diversity within granulomas and across patients [146, 147]. Sawyer et al. [148] introduced the “total cell central preference index” (tCPI), a spatial metric that quantifies whether immune cells are concentrated toward the center or the periphery of granulomas. When combined with immune cell composition data, this allowed stratification of granulomas into distinct recurrent spatial patterns, observed across most TB patients. Whether tCPI can predict disease outcome or treatment response in TB remains an open question to be investigated.

#### 3.3.2 COVID‐19

During the COVID‐19 pandemic, accelerated adoption of spatial technologies enabled rapid insights into disease pathology. Early studies focused on establishing what was happening in the infected lung. Desai et al. [149] were among the earliest to apply spatial transcriptomics to autopsy lung samples and revealed heterogeneous immune responses, with IFN‐driven gene expression localized to areas of active viral infection, which have been shown to be nonuniformly distributed in the lungs [150]. Rendeiro et al. [151] generated one of the first spatial lung atlases using a 36‐marker IMC panel, showing disrupted tissue architecture and widespread immune infiltration. Balachander et al. [152] used TSA‐based multiplex IF to map proinflammatory cytokine expression directly to pulmonary macrophages and alveolar epithelial cells, linking cellular identity to spatial position within the inflamed tissue. Most strikingly, Ng et al. [153] compared gut and lung immune microenvironments in the same patients, revealing that while lymphoid architecture was preserved in the gut, it was disrupted in the lung, suggesting that the failure to maintain organized immune structures may be a key feature of severe pulmonary disease rather than a consequence of it.

#### 3.3.3 HIV

In HIV, the virus persists in tissue reservoirs such as the lymph nodes, gut, or brain. Understanding these niches has become a major focus [13, 154]. Papadopoulos et al. [155] combined multispectral imaging and GeoMx DSP (a sequencing‐based spatial omics platform) to show that HIV persistence in B‐cell follicles is maintained by a local immunoregulatory environment, where follicular helper T cells suppress CD8+ cytolytic activity, allowing viral reservoirs to persist. This spatial logic, a structured tissue compartment that restricts cytotoxic access, echoes what has been described in tumor immunology, where CD8+ T cell exclusion from tumor cores is a defining feature of immune evasion in some types of cancer [156, 157].

While TB, COVID‐19, and HIV are the most studied infectious diseases using spatial approaches, particularly spatial transcriptomics, recent efforts are expanding into spatial multiomics and into studying other pathogen‐related diseases [158, 159, 160, 161, 162].

### 3.4 Immunometabolism

The field of immunometabolism explores the dynamic relationship between immune responses and metabolic processes, ranging from systemic inflammation in metabolic disorders to cell‐intrinsic metabolic reprogramming of immune cells during disease. Immune function is shaped not only by which cells are present but by where they are and how they are organized, and in immunometabolism, a parallel dimension emerges: the metabolic state of immune cells itself is spatially determined [163].

#### 3.4.1 Obesity and Adipose Tissue Inflammation

Obesity is a common comorbidity across many diseases and significantly impacts immune–metabolic dysregulation [164]. Research has focused on identifying which immune cells accumulate in adipose tissue and how their metabolic states influence their function.

A hallmark of inflamed adipose tissue is the formation of crown‐like structures (CLS). CLS, much like granulomas in infectious disease, represent spatially organized immune responses built around a local danger signal. These histological structures are clusters of macrophages that surround dead or dying adipocytes, which in mouse models have been suggested to be of a size that prevents normal clearance, triggering a locally confined proinflammatory response [165]. Within these structures, spatially resolved analysis has revealed distinct macrophage subpopulations with divergent metabolic profiles whose interaction with local fibroblasts seems to determine whether the tissue resolves inflammation or progresses toward fibrosis, a major complication in obesity [166, 167].

#### 3.4.2 Cancer and Metabolic Niches

The tumor microenvironment is not only immunologically diverse but also metabolically heterogeneous [168, 169]. In high‐grade serous ovarian cancer, Berrell et al. [170] used a high‐plex imaging panel including metabolic markers such as iNOS, GLUT1, IDO1, G6PD, and citrate synthase, among others, to characterize distinct metabolic niches. Their findings linked low metabolic activity with higher Treg proximity and worse survival, suggesting that tumor metabolism shapes immune infiltration patterns.

While spatial transcriptomics and proteomics technologies offer insights into metabolic regulation, the number of spatial studies in this field remains limited. This may partly reflect the fact that many metabolism‐focused researchers turn to spatial metabolomics, which enables a direct mapping of metabolite distribution. However, spatial metabolomics still trails behind spatial transcriptomics and proteomics. New integrative studies have begun to bridge this gap, introducing strategies that combine IMC and MS imaging data to simultaneously capture cellular phenotypes and metabolic function within the same spatial context [171].

### 3.5 Neuroimmunology

Microglia are the tissue‐resident immune cells of the brain and play central roles in both neuroprotection and neuroinflammation. They are deeply implicated in neurodegenerative diseases like multiple sclerosis, Parkinson's disease, and Alzheimer's disease [14, 172].

Ramaglia et al. [173] were one of the earliest to demonstrate the potential of high‐plex imaging in the brain, showing that IMC could discriminate between resident microglia and recruited immune cell populations within multiple sclerosis lesions, map their spatial relationships to lesion type and activity, and bring quantitative spatial analysis to the study of brain lesion architecture.

In the context of Alzheimer's disease, a spatial proteomics study has shown that microglia span a continuum of immune activation states shaped by local tissue environments, rather than discrete “resting” or “activated” states. This spatially resolved view highlights how microglial phenotypes are dynamically regulated by their microenvironment and become skewed toward a dysfunctional phenotype in diseased regions [174]. Previous spatial studies have further shown that chronic proximity to pathological deposits, such as amyloid β‐plaques, progressively drives microglia toward dysfunctional states [175, 176, 177]. Such spatially resolved insights could inform therapeutic strategies in Alzheimer's disease to go beyond amyloid clearance and address the functional state of microglia within their local tissue niches.

As the immune activity in the central nervous system is increasingly recognized as region‐specific, spatial biology provides a critical framework to map stromal, glial, and immune cell interactions [174, 178].

### 3.6 Regenerative Immunology

Beyond its role in defense, the immune system is increasingly recognized as a central orchestrator of tissue repair and regeneration. The field of regenerative immunology seeks to understand how immune cells coordinate pathogen clearance, debris removal, and the establishment of a prohealing microenvironment following injury [179]. In acute myocardial infarction, Wünnemann et al. [37] observed monocyte recruitment concentrated in a defined endocardial hub driven by locally upregulated molecular signals. Disrupting this spatially confined recruitment worsened cardiac function, demonstrating that the organization of the immune response in space could determine healing outcome. A parallel principle emerges in periodontal disease. Theofilou et al. [180] showed that in healthy gingiva, neutrophils are spatially confined, maintaining homeostasis, in contrast to other contexts where neutrophil infiltration suggests inflammation. This spatial confinement may be key to facilitate effective bacterial clearance and avoid disease progression [181, 182].

The application of high‐dimensional spatial proteomics in regenerative settings remains limited, highlighting an important opportunity to further define how immune cell positioning and interactions within tissue niches govern successful tissue repair.

### 3.7 Transplant Immunology

Allogeneic transplantation triggers immune recognition of donor tissue, particularly due to differences in major histocompatibility complex (MHC) molecules. The recipient's T cells recognize these MHC molecules as foreign, activating a cascade that can result in graft rejection [183]. Because rejection unfolds within the anatomical context of the graft, spatial profiling of the donor‐recipient interface yields deeper insights into transplant immunopathology [184, 185, 186].

In liver transplantation, Zhou et al. [187] integrated single‐cell, spatial transcriptomics and multiplex immunofluorescence across multiple stages and observed that transplant‐associated T cells driving rejection are not diffusely distributed but concentrated in defined tissue niches. Similarly, in a study of kidney allograft biopsies, Alexander et al. [188] showcased an application of IMC, AI‐based classifiers and spatial metrics to distinguish between different types of rejection based on the spatial arrangement of immune cells within glomeruli and tubules. Integrating molecular spatial data into established systems such as the Banff classification, the traditional diagnostic framework in kidney transplantation, could help uncover mechanisms underlying rejection, improve diagnostic accuracy, enhance allograft monitoring, inform intervention strategies, and ultimately move the field closer to achieving long‐term immune tolerance [189].

## 4 Roadblocks and New Routes in Spatial Immunity

The rapid advancement of spatial biology applied to immunity is accompanied by a set of unresolved challenges at the research level that could also hinder clinical adoption, as well as emerging opportunities that promise to redefine our understanding of in situ immunology. Here we address the key technical and analytical hurdles currently limiting the field and explore emerging frontiers that are beginning to reshape spatial immunity.

### 4.1 Technical and Analytical Hurdles

#### 4.1.1 Sample Preparation, Staining, and Antibody Validation

Despite the availability of standardized protocols from commercial platforms, in practice assay development often requires substantial optimization. A significant bottleneck is the limited repertoire of well‐validated antibodies, which restricts panel design in antibody‐based platforms. Moreover, the availability of probes (antibodies/oligonucleotides) may vary over time depending on batch performance or commercial supply changes, potentially requiring panel redesign and revalidation [190]. While custom panels can be developed, they often vary across laboratories, complicating reproducibility. Efforts from researchers to publish detailed, open‐access staining protocols are therefore invaluable in promoting transparency, reproducibility, and community standards [62, 191, 192, 193, 194, 195].

#### 4.1.2 Throughput and Multiplexing Trade‐Offs

Imaging‐based spatial proteomics technologies offer single‐cell resolution but are limited in the number of measurable markers compared with sequencing‐based spatial transcriptomics. This constraint largely stems from their reliance on antibody‐based detection, which introduces challenges such as fluorescence spectral overlap and progressive loss of tissue and antigen integrity caused by the iterative staining and detection cycles required. As platforms evolve, improvements in staining chemistries and signal detection are expected to enhance multiplexing. For instance, Chang et al. [196] recently introduced PRISM (profiling of RNA in situ through single‐round imaging), a method that expands multiplexing capacity for spatial RNA imaging not by adding more fluorescent channels or iterative staining cycles, but by exploiting fluorescence intensity gradations within only four channels. Each transcript is assigned a unique combination of intensity levels across channels, read out in a single imaging round using conventional microscopes, enabling up to 64‐plex spatial RNA detection.

#### 4.1.3 Data Preprocessing, Cell Segmentation, and Cell Annotation

Spatial datasets provide an extraordinary depth of information, but this comes at the cost of increased noise and potential technical biases. Therefore, robust data preprocessing steps are essential, as inadequate preprocessing can propagate errors and significantly impact downstream analysis such as cell type annotation [197, 198]. While several strategies are being developed, preprocessing pipelines for spatial datasets remain largely nonstandardized [199, 200, 201, 202].

Inaccurate cell segmentation, particularly in densely packed regions or irregularly shaped cells, can also significantly bias downstream analysis [203]. The gold standard in cell segmentation is based on the creation of expanded cell masks combined with manual marker gating, which is labor‐intensive and error‐prone [198, 201]. Emerging DL methods have substantially improved nuclei and cellular segmentation accuracy and automation [204]. Additionally, ML and DL approaches for cell phenotyping are also being developed to overcome the limitations of traditional strategies, though no universal standard tool has yet been widely adopted [115, 205, 206].

#### 4.1.4 Computational Demands

Spatial datasets are exceptionally large; they can exceed 100 GB per sample. Open‐science initiatives encourage data deposition in public repositories, following guidelines for appropriate publication [105, 119]. However, this raises questions about long‐term storage capacity and accessibility as spatial studies scale globally.

Spatial data analysis is still in its early stages, with laboratories often using different pipelines or developing their own tools. Thus, researchers are increasingly encouraged to share them through open repositories. However, the absence of clear standards for documentation, implementation detail, and usability often limits both reproducibility and adoption.

Further, as analytical frameworks grow in complexity, high performance computing infrastructure is essential particularly for those pipelines that integrate DL‐based methods [204]. Researchers in resource‐limited settings may face barriers to accessing such computational capacity.

#### 4.1.5 Multimodal and Multiomic Data Integration

To capture a holistic view of tissue biology, researchers are increasingly combining multimodal and multiomics data [19, 204, 207]. Some commercial platforms are already capable of combining proteomic and transcriptomic data in a single assay, facilitating data integration and analysis. Integration is more straightforward when modalities share spatial coordinates (e.g., post‐run H&E or IHC after PhenoCycler or Xenium runs in the same tissue sample). For such aligned datasets, algorithmic frameworks and DL models have been developed to merge spatial layers [208, 209, 210].

However, integration becomes substantially harder when data derive from different samples or platforms. Then biological differences and platform‐specific biases must be addressed. Several computational strategies have been developed [211, 212], including new computational frameworks such as SpatialData [92] or DL‐based models [213].

#### 4.1.6 From Bench to Bedside

The high‐plex modality undoubtedly plays a key role in research and is already finding its way into clinical settings, with applications emerging in clinical trials [214, 215]. However, multiplex immunofluorescence panels comprising up to 10 markers remain the most implemented format in clinical contexts [216, 217, 218, 219]. Clinical translation will likely rely on marker reduction strategies to define minimum predictive panels [220, 221]. Until workflows—from sample preparation to data interpretation—become sufficiently streamlined and scalable, high‐plex assays will remain impractical for pathology workflows, where time and workload constraints demand “one‐click” solutions [222, 223].

Beyond workflow optimization, prospective validation of spatial biomarkers for clinical use presents unique technical and regulatory challenges. Unlike conventional biomarkers such as PD‐L1, where scoring algorithms are mathematically explicit and FDA‐approved as companion diagnostics for specific drug‐tumor contexts, spatial biomarkers lack standardized definitions and regulatory precedent [224, 225]. Spatial metrics require defining cell‐to‐cell distances, tissue compartment boundaries, and higher‐order spatial statistics [110]. An instructive intermediate case is the Immunoscore, which quantifies the densities of two immune cell populations, CD3+ and CD8+ T lymphocytes, across two spatially defined tumor regions, the tumor core and the invasive margin, to predict risk of relapse in colorectal cancer [226, 227]. Validated across 14 centers in 13 countries and prospectively confirmed in two Phase 3 clinical trials, it achieved CE‐IVD status in the EU and is available in FDA CLIA‐certified laboratories for routine use [228, 229, 230, 231]. That this level of multicentric prospective effort was required for a metric as conceptually straightforward as cell density underscores the challenge ahead for spatially complex biomarkers. Standardization efforts are nascent, with consensus guidelines for multiplex IHC/IF reporting only recently published [232, 233, 234].

**FIGURE 4:**
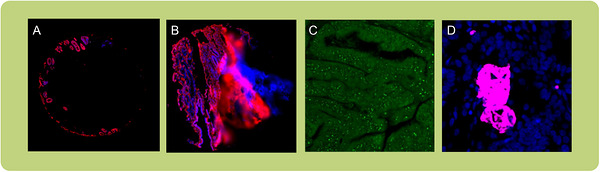
Examples of staining artifacts. (A) Missing TMA core. (B) Detached TMA with blurred imaging. (C) Nonspecific staining and autofluorescence. (D) Staining aggregates and overexposure (figure panels assembled using BioRender.com).

Another important consideration for clinical translation is cost, as high‐plex spatial assays remain expensive on a per‐sample basis in the research setting. In tumor immunology research, the use of tissue microarrays (TMAs) is common to reduce costs and maximize throughput, yet even in this format technical limitations often arise. Reports have shown that within large multiplex panels, certain proteins/RNA fail to stain reliably, and during iterative staining cycles some TMA cores may be physically lost or detached from the slide [235, 236]. These issues compromise data completeness and reproducibility. Moreover, because most of these imaging outputs are fluorescence‐based, they remain susceptible to batch effects introduced by sample type, staining artifacts, imaging conditions, or instrument variability (Figure 4). However, the relationship between diagnostic assay cost and overall treatment expenditure is not straightforward. A comparative cost analysis of the TumorProfiler melanoma project, a multiomics diagnostic platform costing approximately five times more than standard next‐generation sequencing, suggests that investment in advanced multiomics profiling may not necessarily translate into increased overall healthcare expenditure, particularly if it has the potential to improve treatment selection [237, 238]. While spatial proteomics via IMC was included as part of this multiomics diagnostic platform, the specific contribution of spatially derived metrics to treatment recommendations was not reported specifically; dedicated cost‐effectiveness analyses specifically assessing the clinical and economic value of spatial biomarkers as decision‐support tools are currently lacking.

### 4.2 Emerging frontiers in Spatial Immunity

#### 4.2.1 Data Inference

Exciting progress is being made in AI‐powered inference, where molecular information is predicted from standard H&E images [41, 239, 240]. For instance, a recent publication presented *ROSIE*, an AI model that can generate multiplex IF images from paired H&E images [241]. The core principle behind these AI‐powered inference models is that they are trained on seen data, containing known markers, to later predict them in unseen data. However, when certain markers are underrepresented or unevenly distributed in the training data, model performance can become biased, leading to poor prediction accuracy for specific targets. Moreover, because these models only learn from predefined patterns, they are inherently limited in their ability to identify novel or rare cellular populations. Such limitations are intrinsic to current approaches and highlight the need for larger, more diverse, and well‐annotated multimodal datasets to improve model generalization [242].

#### 4.2.2 3D Imaging and Super Resolution

Current spatial technologies operate on two‐dimensional tissue sections, which do not fully capture the 3D microenvironment where they come from. This sampling bias could distort perceived interactions between tissue microenvironment components [243, 244, 245]. Most approaches for 3D imaging involve using thin serial sections for 3D image reconstruction, where usual iterative staining procedures can be applied for staining and later computationally reconstructed into volumetric representations. Some studies have sought to overcome the limitations of thin‐section imaging by increasing section thickness [244] or by employing macrosections combined with techniques of tissue clearing and microscopy modalities like light‐sheet fluorescence microscopy [246, 247, 248, 249]. While they can provide highly detailed insights, immunostaining methods capable of achieving the same level of multiplexing as in 2D tissues are not currently developed [245]. Moreover, the technical complexity makes current 3D workflows difficult to scale for routine use in histopathology settings [244].

In terms of resolution, spatial technologies allow us to map immunity at the 2D single‐cell level, typically at 20× or 40× magnification, where the detection sensitivity is constrained by the antibody–fluorochrome chemistry. Expansion microscopy is a recent methodology to physically expand tissues to enhance resolution without specialized optics [250, 251, 252, 253]. Kang et al. [254] have shown a 23‐plex staining using cyclic staining in enlarged tissues.

Looking forward, 3D and super‐resolution imaging are in their early stages, but they hold the potential to map tissues and organs in ways never before possible.

#### 4.2.3 Spatial Atlases

One of the most promising outcomes of the spatial biology revolution is the development of comprehensive spatial atlases of human tissues in health and disease [51, 255, 256, 257, 258, 259]. These atlases serve as reference frameworks or training data for AI‐driven data inference. The continued evolution of 3D imaging and super resolution microscopy promises to refine spatial fidelity of these atlases.

#### 4.2.4 Next‐Generation Tissue Image Cytometry

Recent years have witnessed remarkable progress in pipeline development for spatial analysis, including the integration of AI‐driven image analysis solutions. Given the intrinsic heterogeneity of biological data, achieving fully automated “one‐click” analytical solutions remains challenging. However, with the aim of democratizing spatial data analysis, an exciting avenue for tissue image cytometry software is the incorporation of AI‐assisted interfaces (interactive chatbots or guided analysis modules) to support nonexpert users and streamline analysis [260].

#### 4.2.5 Advanced Computational Tools for Spatial Omics

As spatial datasets continue to grow in scale, resolution, and multimodal complexity, mathematical innovation in computational frameworks will be a key driver of what spatial biology can reveal. For instance, SPLISOSM (spatial isoform statistical modeling) enables detection of spatially variable transcript isoforms, revealing spatial regulatory mechanisms that cannot be detected when only measuring total gene expression [261]. At the tissue organization level, NicheCompass moves beyond colocalization of cell types to identify tissue niches based on active signaling events occurring between neighboring cells, revealing the communication logic underlying tissue architecture rather than simply describing who is next to whom [262].

## 5 Concluding Remarks

Spatial biology has redefined how we observe immune responses, offering a direct window into how immune cells organize, interact and function within tissues.

Specifically, image‐based spatial platforms serve as a powerful approach allowing for the visualization of immune activity in space. The methodologies and technologies discussed in this review provide valuable insights into understanding immunity across different pathologies.

While spatial technologies are still maturing, they are already shaping the future of diagnostics and therapeutic discovery. Their integration into clinical settings will depend on overcoming current challenges around scalability, standardization, data analysis, and cost.

As spatial biology continues to unfold, it will push immunology, pathology, and precision medicine into a new era of spatially informed care.

## Author Contributions

L.C.‐P. addressed the major reviewers’ comments and contributed to the rewriting, editing, and finalization of the manuscript. S.G.S. drafted the original manuscript and revised all subsequent versions. F.M. and D.Y. contributed technical content and assisted in writing the sections related to spatial biology and imaging technologies. R.C.E. and J.B. conceptualized the study, provided the foundational framework for its development, and critically reviewed all versions of the manuscript. All authors reviewed and approved the final version for submission.

## Funding

L.C.‐P. acknowledges QUT Postgraduate Research Award and ARC CTET Top‐Up Scholarships. J.B. acknowledges NHMRC Ideas grant.

## Ethics Statement

The authors have nothing to report.

## Conflicts of Interest

R.C.E. is the co‐founder and CEO of TissueGnostics GmbH, Austria. F.M. is a Senior Product Manager at TissueGnostics GmbH, Austria. All other authors declare no conflicts of interest.

## Data Availability

The authors have nothing to report.
